# Genome-wide identification of functionally distinct subsets of cellular mRNAs associated with two nucleocytoplasmic-shuttling mammalian splicing factors

**DOI:** 10.1186/gb-2006-7-11-r113

**Published:** 2006-11-30

**Authors:** Margarida Gama-Carvalho, Nuno L Barbosa-Morais, Alexander S Brodsky, Pamela A Silver, Maria Carmo-Fonseca

**Affiliations:** 1 Instituto de Medicina Molecular, Faculdade de Medicina, Universidade de Lisboa, Av. Prof. Egas Moniz, 1649-028 Lisboa, Portugal; 2 Hutchison/MRC Research Centre, Department of Oncology, University of Cambridge, Hills Road, Cambridge CB2 0XZ, UK; 3 Department of Systems Biology, Harvard Medical School, 200 Longwood Ave, Alpert 536, Boston, MA 02115, USA; 4 Department of Molecular Biology, Cell Biology and Biochemistry and Center for Genomics & Proteomics, Brown University, 69 Brown Street, Providence, Rhode Island 02912, USA

## Abstract

**Background:**

Pre-mRNA splicing is an essential step in gene expression that occurs co-transcriptionally in the cell nucleus, involving a large number of RNA binding protein splicing factors, in addition to core spliceosome components. Several of these proteins are required for the recognition of intronic sequence elements, transiently associating with the primary transcript during splicing. Some protein splicing factors, such as the U2 small nuclear RNP auxiliary factor (U2AF), are known to be exported to the cytoplasm, despite being implicated solely in nuclear functions. This observation raises the question of whether U2AF associates with mature mRNA-ribonucleoprotein particles in transit to the cytoplasm, participating in additional cellular functions.

**Results:**

Here we report the identification of RNAs immunoprecipitated by a monoclonal antibody specific for the U2AF 65 kDa subunit (U2AF^65^) and demonstrate its association with spliced mRNAs. For comparison, we analyzed mRNAs associated with the polypyrimidine tract binding protein (PTB), a splicing factor that also binds to intronic pyrimidine-rich sequences but additionally participates in mRNA localization, stability, and translation. Our results show that 10% of cellular mRNAs expressed in HeLa cells associate differentially with U2AF^65 ^and PTB. Among U2AF^65^-associated mRNAs there is a predominance of transcription factors and cell cycle regulators, whereas PTB-associated transcripts are enriched in mRNA species that encode proteins implicated in intracellular transport, vesicle trafficking, and apoptosis.

**Conclusion:**

Our results show that U2AF^65 ^associates with specific subsets of spliced mRNAs, strongly suggesting that it is involved in novel cellular functions in addition to splicing.

## Abstract 2

A genome wide identification of mRNAs that were associated with the splicing factor subunit U2AF65 suggests that U2AF65 associates with specific subsets of spliced mRNAs and may be involved in novel cellular functions in addition to splicing.

## Background

Recent work emphasizes how post-transcriptional control of gene expression is more pervasive than was previously thought. It is now clear that every step of mRNA metabolism can be subject to dynamic regulation events that act in a transcript-specific manner [1], and genome-wide approaches are revealing how post-transcriptional regulation introduces a new layer of control that allows the cell to rapidly activate and coordinate the expression of functionally related sets of genes (for reviews, see Mata and coworkers [2] and Hieronymus and Silver [3]). At the heart of these regulatory events is the mRNP complex, a unique and dynamic combination of proteins (and also small noncoding RNA molecules) that accompanies each particular mRNA from the moment its first nucleotides are synthesized. RNA-binding proteins (RBPs) are the major determinants of the fate of an mRNA and so are the main effectors of the post-transcriptional control of gene expression. RBPs interact with mRNA through the recognition of sequence elements, and the distribution of distinct consensus binding motifs in each mRNA species has been proposed to constitute a combinatorial code that, by interfacing with RBPs, will coordinate the destiny of groups of transcripts in response to the cell's need [1,4]. This coordinated regulation can be simultaneously imposed at different levels, as a growing number of studies depict RBPs as multifunctional proteins that can interface with the different cellular machines that act upon mRNA [1].

The U2 small nuclear (sn)RNP auxiliary factor (U2AF) is a highly conserved heterodimeric essential splicing factor, composed of a 65 kDa and a 35 kDa subunit, with a well characterized role during the early steps of spliceosome assembly [5,6]. The large subunit of U2AF (U2AF^65^) has three RNA recognition motifs (RRMs) that determine its high affinity for the intronic polypyrimidine tract upstream of the 3' splice site [5], whereas the small subunit of U2AF (U2AF^35^) is responsible for the recognition of the conserved AG dinucleotide at the 3' splice site [7-9]. U2AF and the branch point binding protein SF1/mBBP [10] cooperatively establish a transient interaction with the pre-mRNA that is required for the recruitment of the U2snRNP, leading to the subsequent assembly of an active spliceosome complex.

Immunofluorescence microscopy reveals that, at steady state, U2AF is detected exclusively in the cell nucleus [11]. However, both subunits of the heterodimer shuttle continuously between the nucleus and the cytoplasm [12], raising the possibility that U2AF may persist associated with mRNPs in transit to the cytoplasm. Consistent with this view, U2AF was implicated in mRNA export in both mammalian and *Drosophila *systems [13,14]. To address the question of whether U2AF^65 ^associates with mature mRNAs, we performed a genome-wide analysis of transcripts that were immunoprecipitated by a specific monoclonal antibody. For comparison we analyzed mRNAs immunoprecipitated by an antibody directed against the polypyrimidine tract binding protein (PTB), one of the first well characterized paradigms of a multifunctional RBP [15]. PTB was originally identified as a component of splicing complexes [16] and was later shown to be an alternative splicing regulator. PTB binds to pyrimidine-rich tracts on the precursor mRNA and, in general, functions to repress the inclusion of alternative exons [17]. In addition to its role in splicing, PTB has been shown to regulate 3' end processing [18,19] and to be required for internal ribosome entry site (IRES)-mediated translation of several viral and cellular mRNAs [20]. PTB has also been implicated in the regulation of mRNA localization [21] and stability [22,23].

Using a combination of immunoprecipitation and microarray analysis, we identify subsets of mRNAs that associate differentially with U2AF^65 ^and PTB, corresponding to approximately 10% of all cellular mRNAs expressed in HeLa cells. We further demonstrate that U2AF^65 ^binds either directly or indirectly to defined spliced mRNA species, arguing that this splicing protein most likely performs other functions in the cell.

## Results

### U2AF^65 ^is detected in cytoplasmic fractions in association with RNA

**Figure 1:**
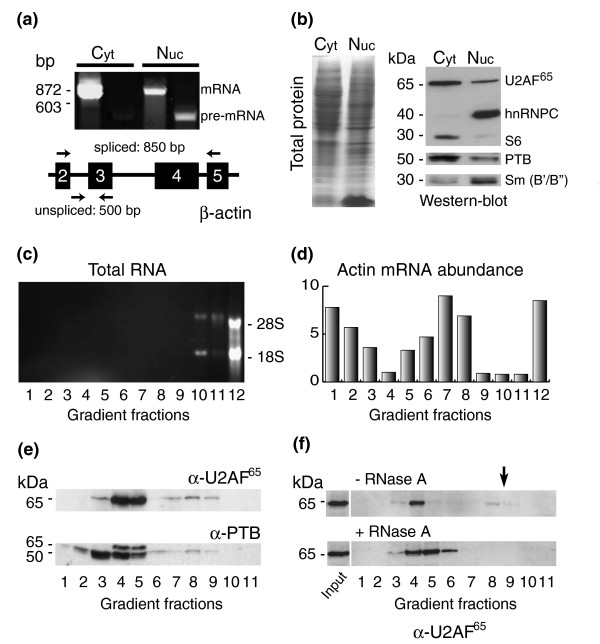
Subcellular distribution of U2AF^65^. **(a) **RT-PCR analysis of spliced and unspliced β-actin mRNA in cytoplasmic (Cyt) and nuclear (Nuc) fractions isolated from HeLa cells. Primer sequences on actin RNA and size of expected amplification products are depicted below the gel. **(b) **Western blot analysis of the cytoplasmic (Cyt) and nuclear (Nuc) fractions using antibodies against the indicated proteins. Molecular weight markers are shown on the left. Coomassie staining of the gel used for blotting confirms that both fractions contain similar amounts of total protein. **(c) **Agarose gel electrophoresis of total RNA extracted after Nycodenz gradient fractionation of cytoplasmic samples. rRNA bands are indicated on the right. Numbers indicate gradient fractions from low to high density. **(d) **Semiquantitative RT-PCR analysis of actin mRNA in the gradient fractions. **(e) **Western blot analysis of gradient fractions using anti-U2AF^65 ^and anti-PTB antibodies. Molecular weight markers are indicated on the left. The 65 kDa band in the membrane after reprobing for PTB corresponds to residual signal from U2AF^65 ^detection. **(f) **Western blot analysis of gradient fractions and input samples using anti-U2AF^65 ^antibody. Extracts were either mock-treated (control) or incubated with RNase A before fractionation. Molecular weight markers are indicated on the left. Arrow points to the intermediate density fraction where U2AF^65^-containing complexes accumulate. PTB, polypyrimidine tract binding protein; RT-PCR, reverse transcription polymerase chain reaction; U2AF, U2 small nuclear RNP auxiliary factor.

To investigate whether U2AF^65 ^associates with mature mRNA complexes, we separated HeLa cell lysates into nuclear and postnuclear cytoplasmic fractions, as previously described [24]. The resulting fractions were characterized by reverse transcription (RT)-polymerase chain reaction (PCR; Figure 1a) and Western blotting (Figure 1b). As expected, the results show that unspliced actin pre-mRNA is detected exclusively in the nuclear fraction, whereas spliced mRNA is enriched in the cytoplasmic fraction (Figure 1a). Moreover, well known nuclear proteins such as hnRNP C and Sm are predominantly detected in the nuclear fraction, whereas ribosomal protein S6 is enriched in the cytoplasmic fraction (Figure 1b). Unexpectedly, a significant proportion of U2AF^65 ^was present in the cytoplasmic fraction, and a similar result was observed for PTB (Figure 1b). Taking into account that immunofluorescence microscopy reveals that both U2AF^65 ^and PTB are highly concentrated in the nucleus, we consider it most likely that the relatively large amounts of these proteins detected in the cytoplasmic fraction reflect leakage of molecules that are not tightly bound in the nucleus.

To determine whether U2AF^65 ^and PTB molecules detected in cytoplasmic fractions are associated with mRNPs, we fractionated the extracts through Nycodenz gradients, which efficiently separate mRNP complexes from free protein and ribosomes [24]. Agarose gel electrophoresis of gradient samples shows that 28S and 18S rRNAs concentrate in fractions 10 to 12 (Figure 1c). Semi-quantitative RT-PCR reveals that actin mRNA is preferentially detected in fractions 1, 2, 7, 8, and 12 (Figure 1d). These results suggest that the Nycodenz gradient resolves at least two distinct pools of mRNA: one pool associates with ribosomes (corresponding to highest density fractions) and the other consists of lower density complexes devoid of ribosomes. Western blot analysis indicates that both U2AF^65 ^and PTB are enriched in lower density fractions (Figure 1e), arguing that these proteins are not major components of polysomes. Treatment of cell lysates with RNase A before gradient fractionation resulted in a shift of U2AF^65 ^from intermediate density fractions (fractions 8 and 9) to less dense regions of the gradient (fractions 4 to 6; Figure 1f), indicating that the presence of the protein in these fractions is mediated by association with RNA. Taken together, these results suggest that U2AF^65 ^associates with RNP complexes that are not tightly bound in the nucleus and therefore are unlikely to contain pre-mRNA.

### Microarray analysis identifies U2AF^65^-associated mRNAs

To further investigate whether U2AF^65 ^associates with postsplicing mRNP complexes in the cell, we performed microarray analysis of mRNAs immunoprecipitated by an anti-U2AF^65 ^antibody. For comparison, we analyzed RNAs immunoprecipitated by an anti-PTB antibody. Three independent immunoprecipitation experiments were performed for U2AF^65 ^and two independent immunoprecipitations were performed for PTB. A mock RNA-immunoprecipitation assay using empty beads was performed in parallel with each experiment. Polyadenylated RNA from the input and immunoprecipitated samples was reverse transcribed, end-tagged, and amplified by PCR within the linear range. The resulting cDNAs were then labeled and hybridized to Affymetrix 133U Plus 2.0 microarrays, which provide comprehensive coverage of the whole transcribed human genome. No significant amplification of cDNA was obtained in any of the mock immunoprecipitations using beads devoid of antibody. From each of the five precipitation experiments, we hybridized two arrays, corresponding to the input RNA and the immunoprecipitated RNA.

**Figure 2:**
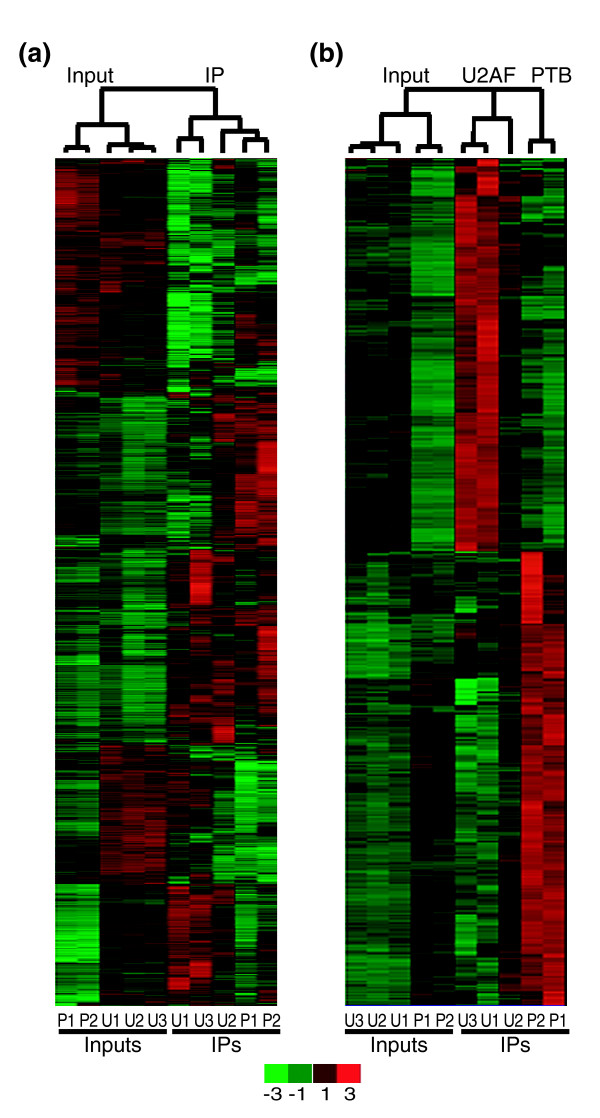
Clustering analysis of microarray data. **(a) **Unsupervised clustering of the microarray dataset was performed with the dChip software using standard settings considering all nonredundant probes with positive hybridization signal. The dataset includes microarray hybridization results from input and immunoprecipitation (IP) samples from three experiments with anti-U2AF^65 ^antibody (U1 to U3) and two experiments with anti-PTB antibody (P1 and P2). Sample clustering defines a tree with two first level branches corresponding to input and IP samples. **(b) **Re-clustering analysis after clearing transcripts that were over-represented either in the inputs or in all immunoprecipitation samples. Sample clustering defines a tree with three first level branches corresponding to input, U2AF^65^, and PTB immunoprecipitation samples. For clustering analysis, the probe signal intensities for each mRNA are standardized to have mean 0 and standard deviation 1 across all samples. The color scale for mRNAs is presented as follows: red represents expression level above mean expression of a gene across all samples, black represents mean expression; and green represents expression lower than the mean. Because of the standardization, probe signal intensities most likely fall within [-3, 3]. PTB, polypyrimidine tract binding protein; U2AF, U2 small nuclear RNP auxiliary factor.

Microarray hybridization data was analyzed using d-Chip software [25]. To obtain an overview of RNA profiles from the input and immunoprecipitation samples we first performed an unsupervised clustering analysis on the complete dataset, corresponding to ten microarray hybridization experiments. Before clustering, a reduced list of transcripts with a viable number of elements for analysis was generated by applying a minimal filter, selecting for probes with an absolute difference in expression level between input samples and immunoprecipitation samples of at least 100 (approximately 15,500 probes out of the 54,000 present in the array). Of these, 9781 were nonredundant probes, considered for clustering of both samples and probes. The output tree produced by this analysis is shown in Figure 2a. This unsupervised clustering of the microarray dataset resulted in a clear separation of input and immunoprecipitated samples, indicating that distinct subsets of transcripts are enriched by the RNA immunoprecipitation, depending on the target antigen. Figure 2b presents a supervised clustering of this dataset, emphasizing the existence of a population of transcripts that exhibit differential enrichment in U2AF^65 ^and PTB immunoprecipitation experiments. To generate this cluster, the transcript list was further filtered with standard dChip settings that select for probes that exhibit wide variation across samples. Irrelevant probe clusters present in the tree generated from this set of 1578 nonredundant probes (clusters of probes enriched in all input or immunoprecipitate samples or enriched in input and immunoprecipitate samples from the same experimental replicates) were then manually cleared, followed by re-clustering of the remaining probes. The tree generated by this approach separates samples into three main branches, consistent with their different origins, and highlights the existence of subsets of transcripts that associate specifically with U2AF^65 ^and PTB.

To identify the subset of cellular mRNA molecules that associate with U2AF^65 ^and PTB, we performed a comparison of the microarray hybridization data from each pair of immunoprecipitate and input samples (Additional data file 1). Using this approach, we derived a list of probes that exhibit a significant increase in signal intensity in at least two replicate immunoprecipitation experiments (Additional data file 2). The results obtained from this analysis indicate that both U2AF^65 ^and PTB associate with about 20% of the mRNAs present in the input samples, and approximately half of the transcripts enriched in the immunoprecipitates are common to both proteins. Thus, mRNAs that associate specifically with either U2AF^65 ^or PTB correspond to about 10% of the total expressed transcripts.

### U2AF^65 ^associates with spliced mRNAs

To validate the microarray data, we selected eight transcripts found specifically enriched in the U2AF^65 ^immunoprecipitates and three transcripts not enriched in these samples; similarly, we selected eight enriched and three nonenriched transcripts in samples precipitated by the anti-PTB antibody. The transcripts selected for analysis were chosen in order to cover both high and low enrichment ratios and representative functional categories (see below).

**Figure 3:**
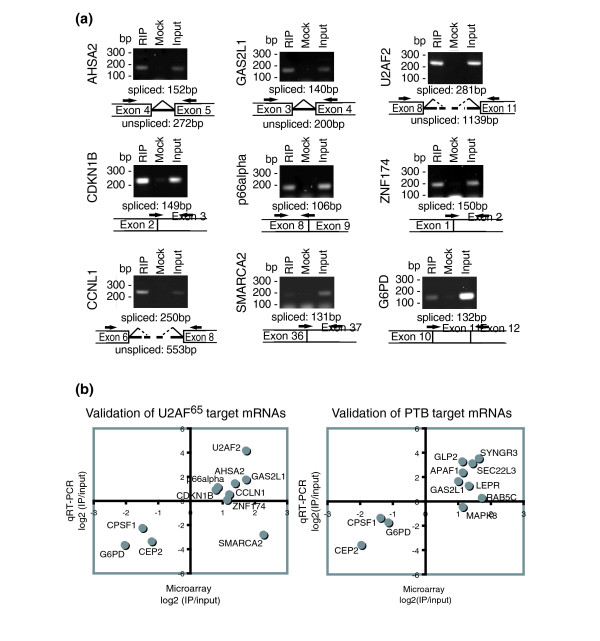
Validation of the mRNA-protein associations identified by microarray analysis. **(a) **RT-PCR amplification of putative U2AF^65 ^associated mRNAs and a nonassociated mRNA (glucose-6-phosphate dehydrogenase [G6PD]) from immunoprecipitation (RIP), mock-imunoprecipitation (Mock), and input samples. For each target, the primer localization and expected size of amplified spliced products is indicated. **(b) **Comparison of microarray quantification and independent real time-PCR quantification of the enrichment index (log2 immunoprecipitation/input) of selected target mRNAs from anti-U2AF^65 ^or anti-PTB immunoprecipitation experiments. Points falling in the positive (+/+) or negative (-/-) sectors of the plot reveal an agreement between microarray and quantitative real-time PCR experiments. The SMARCA2 and MAPK8 mRNA were found to be enriched only in microarray experiments. IP, immunoprecipitation; PTB, polypyrimidine tract binding protein; RT-PCR, reverse transcription polymerase chain reaction; U2AF, U2 small nuclear RNP auxiliary factor.

Three new independent immunoprecipitation experiments were performed using either anti-U2AF^65 ^or anti-PTB antibodies, and the levels of the precipitated mRNAs were analyzed by quantitative real-time PCR. Primers were designed to span splice junctions, and agarose gel electrophoresis confirmed that amplified products from anti-U2AF^65 ^immunoprecipitations corresponded to spliced mRNA species (Figure 3a). Figure 3b depicts a comparison of microarray and quantitative real-time PCR data concerning enrichment of the selected mRNAs in the immunoprecipitated samples. As shown in the figure, the two kinds of data provide the same trends, thus confirming the associations detected by the array experiments.

**Figure 4:**
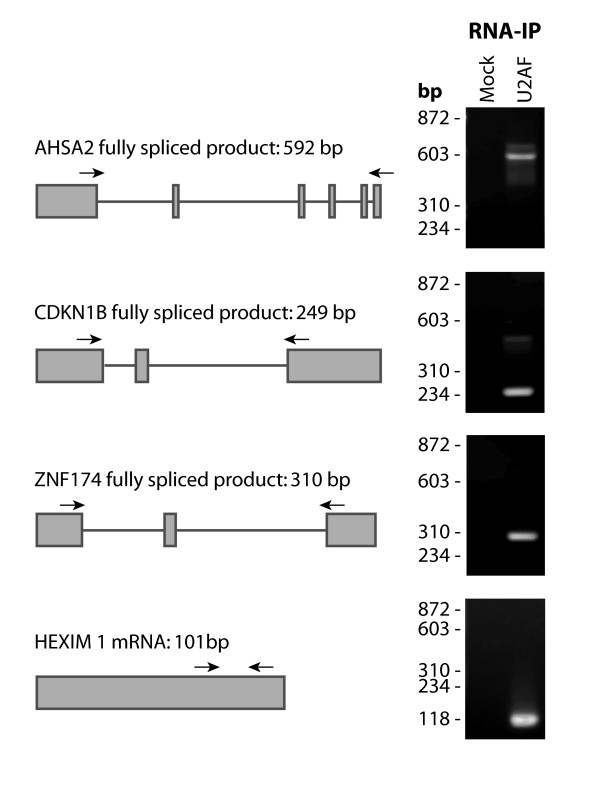
U2AF^65 ^associates with fully spliced mRNAs. Agarose gel electrophoresis of RT-PCR reactions designed to check the splicing status of mRNAs present in control (Mock) and anti-U2AF^65 ^(U2AF) RNA-immunoprecipitations. For each target, the transcript exon/intron structure is represented to scale and primer localization and expected size of fully spliced products are indicated. IP, immunoprecipitation; RT-PCR, reverse transcription polymerase chain reaction; U2AF, U2 small nuclear RNP auxiliary factor.

To confirm that the U2AF^65 ^associated mRNAs are fully spliced, we performed a RT-PCR amplification from the first to last exon for targets with an appropriate size range (Figure 4). As shown in Figure 4, fully spliced mRNAs are detectable in the U2AF^65 ^immunoprecipitates for all the transcripts tested, thus demonstrating the association of U2AF^65 ^with mature mRNA. Transcripts encoded by intronless genes are also found in the anti-U2AF^65 ^immunoprecipitates (Figure 4), suggesting that the association between this protein and mRNA is not splicing dependent.

### mRNAs that associate preferentially with either U2AF^65 ^or PTB belong to different gene ontology groups and have distinct sequence characteristics

To identify the functions of cellular mRNAs that were enriched in the U2AF^65 ^and PTB immunoprecipitated fractions, the probes listed in Additional data file 2 were analyzed using d-Chip software [25] and the DAVID functional annotation tool [26]. The results from Gene Ontology classification reveal a statistically significant bias in the distribution of the products encoded by these mRNAs into different functional categories, when compared with their relative frequency in the genome (Tables 1 and 2). This bias was not observed when analyzing a random list of equivalent size generated from the population of expressed microarray probes. The U2AF^65^-associated mRNA population is highly enriched in mRNAs encoding transcription and cell cycle regulators, whereas PTB-associated mRNAs contain a large proportion of transcripts encoding proteins that are involved in intracellular transport and vesicle trafficking. An additional group of genes related to ubiquitination and signaling through small GTPase molecules exhibits significant enrichment in both U2AF^65^- and PTB-associated mRNA populations. Thus, using two independent software tools to perform the annotation of the mRNAs bound by U2AF^65 ^and PTB, we have obtained evidence for a distinctive functional profile underlying the two populations identified in the microarray analysis.

Next, we performed a systematic sequence analysis for the presence of U2AF^65 ^and PTB consensus binding motifs in the mRNA populations found to be associated preferentially with each of these two proteins. The motifs used for searching the mRNA sequences have previously been described in the literature [7,27] (Additional data file 3). For comparison purposes, we defined for both U2AF^65 ^and PTB a control population of nonassociated transcripts composed of all microarray probes with a negative fold change value in at least two replicate immunoprecipitation experiments (Additional data file 2). To search for the consensus motifs, a Perl script was designed to retrieve the sequence of the longest curated transcript available in the major nucleotide databases that corresponds to the reference sequence for the Affymetrix microarray probe sets belonging to the different transcript subsets (listed in Additional data file 2). We retrieved sequence information for approximately 80% of the transcripts in the original datasets, as listed in Additional data file 4. Because differences in the number of identified consensus motifs will reflect differences in transcript size, the comparison between protein-associated and control mRNA populations was performed after calculating the density of consensus binding motifs for each transcript (number of hits/nucleotide).

**Figure 5:**
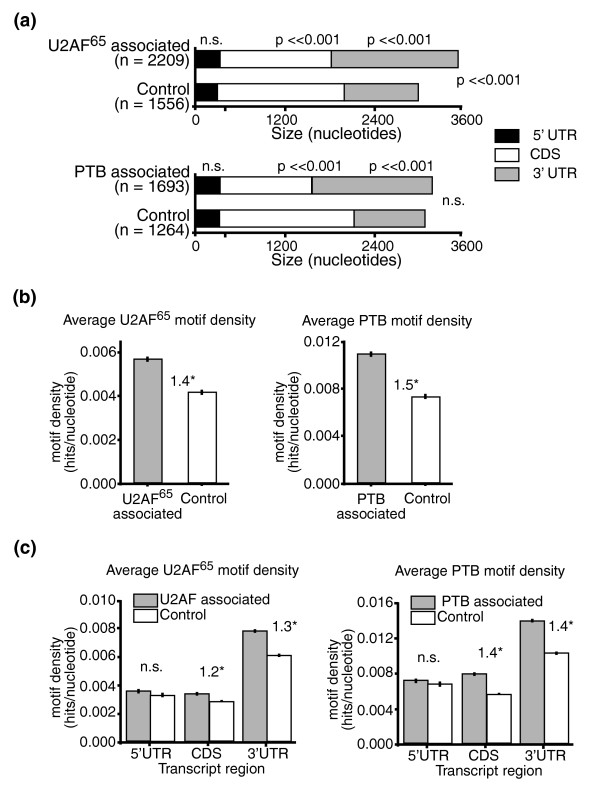
Size analysis of coding sequence and UTRs of U2AF^65^- and PTB-associated mRNA populations. **(a) **Average size of 5' and 3' UTRs and coding sequence (CDS) for mRNAs in the U2AF^65^-associated or PTB-associated populations and their respective control (nonassociated) populations (Additional data file 2). For this analysis, information for the longest curated transcript available in EMBL [40], GenBank [41], and RefSeq [42] databases was retrieved for all entries in each population, when available (Additional data file 4). Statistically significant differences between the associated and the respective control populations are indicated. **(b) **Analysis of putative U2AF^65 ^and PTB binding motifs in selected mRNA populations. The longest curated transcripts for mRNA accessions in the U2AF^65^-associated or PTB-associated populations and their respective control (nonassociated) populations were searched for putative U2AF^65^- and PTB-binding motifs. Graphs present average U2AF^65 ^motif density in the U2AF^65^-associated and control populations, and average PTB motif density in the PTB-associated and control populations. The ratio between values for each associated/control pair is shown. **(c) **Analysis of the distribution of putative U2AF^65^- and PTB-binding motifs in the different transcript regions. Graphs present average U2AF^65 ^motif density by transcript region in the U2AF^65^-associated and control populations and average PTB motif density by transcript region in the PTB-associated and control populations. The ratio between values for each associated/control pair is shown. *P << 0.001. n, population size; n.s., not significant; PTB, polypyrimidine tract binding protein; U2AF, U2 small nuclear RNP auxiliary factor; UTR, untranslated region.

Interestingly, we find that the average size of transcripts in the U2AF^65^- and PTB-associated samples differs from control populations and the variation is mainly due to differences in the average size of the 3'-untranslated region (UTR; Figure 5a). The average 3'-UTR size of the control (nonassociated) population is 60% smaller than the corresponding value for the U2AF^65^- or PTB-associated populations. Comparison between the U2AF^65^- and PTB-associated mRNAs and the respective control nonassociated mRNA population reveals 1.4-fold and 1.5-fold differences in the average density of putative binding sites for these proteins per transcript (Figure 5b). Moreover, comparative analysis of the distribution of putative binding sites in the coding and noncoding regions of the transcripts reveals a statistically significant enrichment in consensus motif density in the coding region and 3'-UTR, contrasting with the 5'-UTR (Figure 5c). These results implicate the coding region and 3'-UTRs in U2AF^65^- and PTB-associated mRNAs as potential targets for the direct binding of U2AF^65 ^and PTB.

## Discussion

Currently available data suggest that a multifunctional nature is more likely to be the rule than the exception among RBPs [1]. Dissecting the cellular roles of multifunctional regulators of gene expression through classical approaches involving knock-down or over-expression is not an easy task, because the inhibition of upstream functions such as transcription and splicing will obscure downstream effects on export, stability, translation, or localization. We therefore decided to take a different approach, beginning with the identification of U2AF^65^-mRNA targets as a starting point to search for potential novel functions of U2AF^65^. Putative U2AF^65 ^mRNA targets identified through the microarray analysis of RNA-immunoprecipitations performed with a monoclonal antibody specific for this protein were confirmed by independent RT-PCR analysis, arguing for the specificity of the results obtained. However, as an additional methodological control for the reliability of this type of large scale approach, we performed a parallel analysis for PTB.

PTB was selected for two main reasons. First, PTB and U2AF^65 ^have relatively similar RNA-binding preferences for pyrimidine-rich stretches, which is underscored by the fact that they can compete for binding to the same intronic sequence elements in alternative splicing regulation [27]. However, in spite of this similarity, we found the mRNA-binding profiles of these two proteins to be quite different. Second, PTB is a well characterized multifunctional RBP that has post-splicing functions on known mRNA targets. Importantly, most of these have been identified as associating with this protein in our large-scale screen.

PTB was implicated in the stabilization of mRNAs encoding secretory granule proteins both in pancreatic islet cells promoting insulin secretion in response to glucose stimulation [22] and during T-lymphocyte activation [28]. Among the PTB targets described in those studies, only two (synaptobrevin 1 and 2) are expressed in HeLa cells, and both were identified as PTB-associated in our screen. A role for PTB as an internal ribosome entry segment (IRES) *trans*-acting factor has also been demonstrated for seven cellular mRNAs [29]. Six of the seven reported mRNAs showed detectable expression levels in our assay. From these, three (Apaf-1, Bag-1, and Myb) were identified as PTB-associated in our screen and another two (IGF1R and Mnt) were positively enriched in the immunoprecipitated samples but were just below the established cut-off values in one of the experiments. It is noteworthy that Apaf-1 and Bag-1 proteins are essential for the apoptotic process (for review, see Spriggs and coworkers [29]). Thus, in addition to having identified previously described PTB mRNA targets, our analysis reveals many novel putative PTB-associated mRNAs that are involved in the same cellular pathways previously associated with this protein (intracellular transport, vesicle trafficking, and apoptosis). The consistency of the results obtained in the analysis of the anti-PTB RNA-immunoprecipitation experiments strongly supports our interpretation of the data obtained for U2AF^65^.

Our results reveal that, in addition to binding to intronic sequence elements in the pre-mRNA during the first steps of spliceosome assembly, U2AF^65 ^also associates with a subset of spliced cellular mRNAs. Functional annotation of the mRNAs that associate specifically with U2AF^65 ^shows significant enrichment in molecules encoding transcription, chromatin, and cell cycle regulators, in particular those involved in the G_1_/S transition. According to the post-transcriptional operon model [4], this bias suggests that the expression of a subset of genes involved in cell cycle progression and in the regulation of specific gene expression programs could be coordinated at the post-transcriptional level by U2AF^65^. Interestingly, a function in cell cycle progression and maintenance of chromatin and nuclear structure has previously been proposed for the *Schizosaccharomyces pombe *homolog of the U2AF large subunit [30]. Based on our findings, we suggest that U2AF^65 ^should be viewed as a multifunctional protein and propose the existence of novel functions for this protein in mRNA metabolism. A precedent for this was established in a recent analysis of a temperature-sensitive RNA binding mutant of the yeast U2AF^65 ^homolog in transgenic *Drosophila *[14], in which this protein was shown to be required for the export of several intronless mRNAs, by directly binding to the message. However, analysis of the U2AF^65^-associated mRNA population that we report in this work did not reveal any bias toward intronless gene transcripts when compared with randomly selected populations mRNA populations (data not shown).

Involvement of shuttling splicing factors in several distinct postsplicing activities has already been reported for members of the U2AF-related SR protein family, which have been shown to participate in mRNA export, translation, and stability [31-33]. Future studies will clarify whether the U2AF^65^-mRNP complexes identified in this work correspond predominantly to nuclear mRNPs in transit to the cytoplasm or whether this protein is also involved in the cytoplasmic metabolism of mRNAs.

## Conclusion

The microarray analysis of α-PTB and α-U2AF^65 ^RNA immunoprecipitations reveals that these splicing factors can associate with a large subset of spliced cellular mRNAs. The mRNA populations associated with each protein contain distinctive sequence features, which are predicted to mediate direct protein-RNA interactions. Additionally, the products encoded by each mRNA population exhibit differential enrichment in proteins that are functionally related. This supports the existence of post-transcriptional regulatory networks involving the specific binding of U2AF^65 ^and PTB to two distinct groups of functionally related mRNAs, similar to what has been recently proposed for other RNA binding proteins [34-36]. Furthermore, our data provide the first clues to novel roles of human U2AF^65 ^in the postsplicing regulation of mRNAs important for transcriptional control and the cell cycle.

## Materials and methods

### Cell culture and extracts

For isolation of mRNP complexes, suspension HeLa cells were grown in Dulbecco's modified Eagle's medium (DMEM), 10% fetal calf serum (FCS) and penicillin/streptomycin, and split 1:2 the day before harvesting. For all other experiments, adherent HeLa cells (ECACC 93021013) were grown in minimum essential medium (MEM), 10% FCS, and nonessential aminoacids at 37°C. Postnuclear cytoplasmic extracts were obtained as described previously [24], using 5 × 10^7 ^cells/ml lysis buffer. For Western blotting analysis, 2.5 μl of extract was run in a 12% SDS-PAGE gel. The pellet fraction was re-suspended in SDS-PAGE buffer and an equivalent volume was analyzed. For RNAse treatment, samples were incubated with 200 μg RNAse A for 15 min at 37°C before gradient fractionation.

### Antibodies

The following antibodies were used for immunoprecipitation or Western blotting: anti-U2AF^65 ^MC3 monoclonal antibody [11], anti-PTB Bb7 monoclonal antibody (American Type Culture Collection, Manassas, VA, USA), anti-Sm Y12 monoclonal antibody [37], anti-hnRNPC1/C2 mAb 4F4 [38], and anti-S6 rabbit polyclonal serum (Cell Signaling Technology Inc, Danvers, MA, USA).

### Isolation of mRNP complexes

mRNP complex isolation by gradient fractionation through a 20% to 60% Nycodenz gradient (Accurate Chemical and Scientific Corp., Westbury, NY, USA) was performed as described previously [24]. After centrifugation, 0.5 ml fractions were collected by underlaying with a 65% Nycodenz solution using an automatic fractionator (Bio-Rad Laboratories, Hercules, CA, USA). Gradient fractions were TCA precipitated, and one-third of the final volume was used for Western blotting analysis. For RNA isolation, fractions were diluted with 250 μl of water and extracted with acid phenol, followed either by denaturing agarose gel analysis or reverse transcription and PCR amplification as described below.

### RNA immunoprecipitation

Isolation of U2AF^65^- or PTB-associated mRNAs under native conditions was performed by immunoprecipitation from 200 μl of precleared HeLa cell lysate, using the anti-U2AF^65 ^MC3 monoclonal antibody (mAb) or the Bb7 anti-PTB mAb for 2 hours at 4°C. Antibody concentrations were adjusted empirically. Immune complexes were precipitated with a slurry with 50% of protein A/protein G agarose beads (GE Healthcare UK Ltd (formerly Amersham Biosciences Corp.), Little Chalfont, Buckinghamshire, England) and blocked with 100 μg/μl of tRNA and RNAse free bovine serum albumin (Ambion, Inc., Austin, USA) by rotating for 1 hour at 4°C. Washes were performed with lysis buffer. Complexes bound to the beads were eluted with TES buffer (10 mmol/l Tris, 0.5 mol/l EDTA, 0.5% SDS [pH 8.0]) by heating at 65°C for 10 minutes. A 15 μl sample of the eluted complexes was used for Western blotting to confirm the efficiency of immunoprecipitation and the rest was Trizol extracted for mRNA analysis.

### Oligonucleotide array expression analysis of U2AF^65^- and PTB-associated mRNAs

Polyadenilated RNAs obtained by anti-U2AF^65 ^and anti-PTB immunoprecipitation were reverse transcribed and PCR amplified using the Super SMART PCR cDNA synthesis kit from Clontech (Clontech Laboratories, Inc., Mountain View, CA, USA), following the manufacturer's instructions. One microgram of input or precipitated RNA or an equivalent sample volume from mock pull-down assays was used was used per RT-PCR reaction to produce samples for microarray hybridization. For each set of samples (input, pull-down, and mock pull-down), a 100 μl test PCR reaction was performed in accordance with the manufacturer's instructions and analyzed by agarose gel electrophoresis to select ideal conditions for amplification in the linear range. For microarray hybridization, 15 μg of PCR amplified cDNA from input and immunoprecipitated samples were prepared and hybridized to Affymetrix GeneChip Human Genome U133 Plus 2.0 Arrays (Affymetric, Inc., Santa Clara, CA, USA), as described elsewhere [39]. Microarray data have been deposited in the GEO database and are available through the series accession numbers GSE6021 (PTB immunoprecipitation data) and GSE6022 (U2AF immunoprecipitation data).

Analysis of microarray results was performed with dChip [25]. To identify mRNAs enriched by the immunoprecipitation procedure, a comparison analysis was performed between each experimental pair dataset (input and immunoprecipitation samples) with output of all genes. Fold change cutoff criteria was established by determining the 95th percentile of the frequency distribution MBEI (model based expression index) increment introduced by experimentally measured standard errors (SE) for all P probes in each experimental dataset pair. Comparison results were combined to identify probe sets satisfying the cutoff criteria (specifically, fold change above the determined cutoff value [using the 90% lower boundary interval] and difference in MBEI > 100) in at least two experiments.

### cDNA synthesis, RT-PCR, and quantitative real-time PCR

cDNA was synthesized from 500 ng purified RNA or one-quarter of Nycodenz gradient fractions using oligo-dT primers and the Superscript II Reverse Transcriptase (Invitrogen Corporation, Carlsbad, CA, USA), as recommended by the manufacturer. PCR amplification was performed using the Bio-X-Act DNA polymerase (Bioline Ltd, London, UK), as recommended by the manufacturer, using the following conditions: 2 minutes at 95°C and 20 to 30 cycles at 95°C for 1 minute, at 55°C for 1 minute, and at 72°C for 1 minute. Primer sequences were as indicated in Additional data file 5. Quantification of ethidium bromide signal from agarose gels was performed on a Typhoon Laser Scanner using the ImageQuant 5.2 software (GE Healthcare UK Ltd, Little Chalfont, Buckinghamshire, England). Real-time quantitative PCR was performed on an ABI PRISM 7000 Sequence Detection System (Applied Biosystems, Foster City, CA, USA) using the SYBR Green PCR master mix (Applied Biosystems, Foster City, CA, USA). Quantification of results was performed with the AbiPrism 7000 SDS software (Applied Biosystems, Foster City, CA, USA). For the quantitative analysis of RNA immunoprecipitations, a standard curve obtained from serial dilutions of Hela cDNA was used to determine the relative amount of specific target RNAs from the different samples.

### Sequence analysis

A Perl script was written to annotate the mRNA sequences and search them for binding motifs. Each mRNA accession number from the Affimetrix array was used to search UniGene (version 176) [14] for a corresponding gene identifier. For each gene, the longest curated transcript available in EMBL [40], GenBank [41] and RefSeq [42] databases was retrieved using Bioperl (version 1.4) modules [43]. For each of these transcripts, the annotation of coding sequence and UTRs was also performed. The transcript sequences were then searched for putative binding motifs for PTB and U2AF using scoring matrices. The sequence YYYYTCTTYYYY was searched for as a putative motif for PTB [44,45], using a scoring matrix (see Additional data file 3). For U2AF a frequency matrix was derived, based on sequences from a SELEX experiment described by Wu and coworkers [46] (Additional data file 3). In all cases, the selected cut-off scores for a positive hit are the highest possible values that produce a Gaussian distribution of the frequency of motifs found in the full-length mRNA.

## Additional data files

The following additional data are available with the online version of this paper. Additional data file 1 is a figure presenting detailed results regarding the initial steps in the microarray data analysis. Additional data file 2 includes tables that contain lists of mRNAs associated with U2AF^65 ^and PTB and control nonassociated mRNA populations. Additional data file 3 is a document detailing the methods used for sequence analysis of consensus binding motifs. Additional data file 4 includes lists of the subsets of mRNAs that were subject to sequence analysis of consensus binding motifs and details the results of this analysis. Additional data file 5 is a document containing the primer sequences used.

## Supplementary Material

Additional data file 1 Figure presenting detailed results regarding the initial steps in the microarray data analysis. Click here for file

Additional data file 2 Lists of mRNAs associated with U2AF^65 ^and PTB and control nonassociated mRNA populations. Click here for file

Additional data file 3 Methods used for sequence analysis of consensus binding motifs. Click here for file

Additional data file 4 Lists of the subsets of mRNAs that were subject to sequence analysis of consensus binding motifs and the results of this analysis. Click here for file

Additional data file 5 Primer sequences used. Click here for file

## Figures and Tables

**Table 1:** Summary of dChip Gene Ontology classification of target mRNA populations

Cellular function	Gene Ontology group	U2AF (%)	PTB (%)	Array (%)
Gene expression	DNA binding	20.7	-	13.7
	Transcription	16.9	-	11.9
	Transcription regulator	13.5	-	8.5
	Transcription factor	9.9	-	6.3
	RNA binding	5.2	-	3.6
Cell cycle	Cell cycle	6.7	-	4.6
	Regulation of cell cycle	4.1	-	2.7
Ubiquitination	Ubiquitin conjugating enzyme activity	1.6	1.7	0.5
Signaling	Small GTPase mediated signal transduction	2.7	3.5	1.5
Intracellular transport and vesicles	Intracellular protein transport	-	5.6	2.5
	Vesicle-mediated transport	-	3.9	2.1
	Golgi apparatus	-	3.6	1.9
	Golgi trans face	-	0.5	0.1

In the U2AF and PTB columns, '-' indicates that enrichment was not significant relative to the proportion of array genes belonging to the Gene Ontology group (*P *> 0.001). PTB, polypyrimidine tract binding protein; U2AF, U2 small nuclear RNP auxiliary factor.

**Table 2:** Summary of DAVID Gene Ontology classification of target mRNA populations

Cellular function	Gene Ontology group	U2AF (%)	PTB (%)
Gene expression	Transcription, DNA-dependent	13.1	10.6
	Transcription from Pol II promoter	3.8	3.1
	Negative regulation of transcription from Pol II promoter	0.7	-
	DNA packaging	1.4	-
	Nuclear mRNA splicing, via spliceosome	1.2	-
	Translation	1.1	-
Cell cycle	Cell proliferation	6.5	5.6
	Cell cycle	4.9	4.1
	Regulation of cell cycle	3	2.3
	G_1_/S transition of mitotic cell cycle	0.6	-
	Mitotic cell cycle	1.3	-
Apoptosis	Death	-	3
	Apoptosis	-	2.8
	Negative regulation of apoptosis	0.9	
Ubiquitination	Ubiquitin cycle	4.9	4.5
	Protein ubiquitination	2.6	2.1
Signaling	Intracellular signaling cascade	6	6.8
	Small GTPase mediated signal transduction	1.9	2.6
Intracellular transport and vesicles	Establishment of localization	-	10.4
	Endosome organization and biogenesis	-	0.4
	Endosome transport	-	0.3
	Vesicle-mediated transport	-	2.1

In the U2AF and PTB columns, '-' indicates that enrichment was not significant relative to the proportion of array genes belonging to the Gene Ontology group (*P *> 0.001). PTB, polypyrimidine tract binding protein; U2AF, U2 small nuclear RNP auxiliary factor.
