# Supplementary material for: Genome-wide identification of functionally distinct subsets of cellular mRNAs associated with two nucleocytoplasmic-shuttling mammalian splicing factors
Source: Genome Biol. 2006 Nov 30;7(11):R113. doi: 10.1186/gb-2006-7-11-r113 (PMC1794580; doi:10.1186/gb-2006-7-11-r113)
Supplement: Additional data file 1 — Figure presenting detailed results regarding the initial steps in the microarray data analysis. [file gb-2006-7-11-r113-S1.pdf]

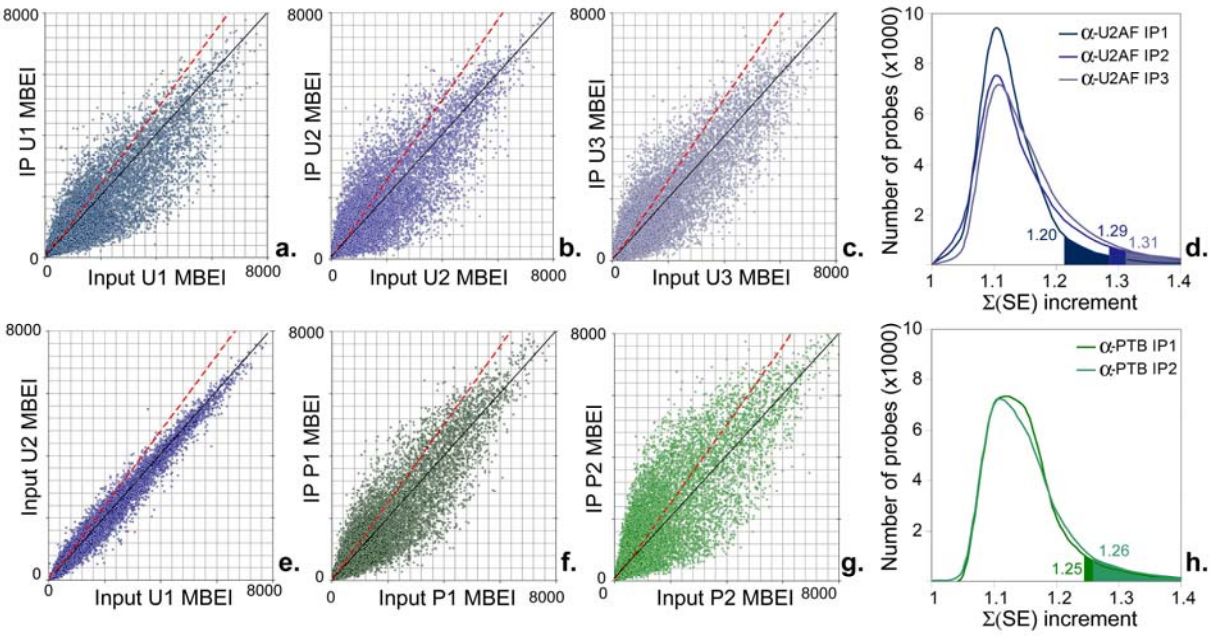

Sup. Figure 1. Comparative analysis of microarray dataset pairs. To establish cut-off criteria for the identification of mRNAs enriched by anti-U2AF^{65} and anti-PTB immunoprecipitation experiments, a comparative analysis of signal intensities for all probes in each input/precipitate sample pair was performed and the frequency distribution of maximum positive variation in probe signal that can be introduced by measured experimental errors, in each experiment was analyzed. **a. to c.** Comparison of the model based expression index (MBEI), a measure of probe signal intensity, for each pair of microarray hybridization data (IP and input) from the three anti-U2AF^{65} RNA-immunoprecipitation experiments (U1 to U3). **d.** Frequency distribution of the increment to the baseline signal of each probe (input MBEI) introduced by measured standard errors in the microarray dataset pair corresponding to an immunoprecipitation experiment (input MBEI SE + immunoprecipitation MBEI SE). The three curves correspond to each of the three anti-U2AF^{65} RNA-immunoprecipitation experiments (α-U2AF IP1 to 3). The 95th percentile of each distribution, marked on the curves, was used as the positive fold change value above which a probe is considered to be enriched by the immunoprecipitation assay. **e.** Comparison of the model based expression index (MBEI) values from the microarray data for the input samples of the α-U2AF IP1 and 2 experiments. Unlike immunoprecipitation sample pairs, independent input samples show a very strong correlation for probe intensities. Application of the established cut-off to this dataset pair results in the selection of less than 10% of the mRNAs found to be positive for enrichment in the analysis of true experimental pairs. **f. and g.** Comparison of probe signals for each pair of microarrays (IP and input) from the two anti-PTB RNA-immunoprecipitation experiments (P1 and P2) as in panels a. to c. **h.** Frequency distribution of the increment to the baseline probe signal introduced by measured standard errors for the two anti-PTB RNA-immunoprecipitation experiments (α-PTB IP1 and 2) as in panel d. The 95th percentile of each distribution is shown. The set of probes found to be present in all arrays in the dataset was used for all comparisons (approximately 18 500 probes). Black line: x=y. The dashed red line represents the cut-off for enrichment used for each experimental pair.
