# Supplementary material for: Genome-wide identification of functionally distinct subsets of cellular mRNAs associated with two nucleocytoplasmic-shuttling mammalian splicing factors
Source: Genome Biol. 2006 Nov 30;7(11):R113. doi: 10.1186/gb-2006-7-11-r113 (PMC1794580; doi:10.1186/gb-2006-7-11-r113)
Supplement: Additional data file 3 — Methods used for sequence analysis of consensus binding motifs. [file gb-2006-7-11-r113-S3.doc]

**Scoring matrices for sequence analysis**

The sequence YYYYTCTTYYYY was searched for as a putative motif for PTB (Perez et al., 1997; Singh et al., 1995a), using the following scoring matrix (where *i* is the position of nucleotide *a*):

| *s­_i_(a)* | | | | | | | | | | | | |
| --- | --- | --- | --- | --- | --- | --- | --- | --- | --- | --- | --- | --- |
| *a i*-> | 1 | 2 | 3 | 4 | 5 | 6 | 7 | 8 | 9 | 10 | 11 | 12 |
| T | 0.5 | 0.5 | 0.5 | 0.5 | 1 | 0 | 1 | 1 | 0.5 | 0.5 | 0.5 | 0.5 |
| C | 0.5 | 0.5 | 0.5 | 0.5 | 0 | 1 | 0 | 0 | 0.5 | 0.5 | 0.5 | 0.5 |

Any 12-mer for which $S_{12}=\sum_{i=1}^{12} s_{i}(a)$ > 5.5 was considered a putative binding site for PTB.

For U2AF a frequency matrix was derived, based on sequences from a SELEX experiment described in (Wu et al., 1999a) (where *i* is the position of nucleotide *a*):

| *a* *i*-> | 1 | 2 | 3 | 4 | 5 | 6 | 7 | 8 | 9 |
| --- | --- | --- | --- | --- | --- | --- | --- | --- | --- |
| A | 2 | 6 | 1 | 5 | 1 | 1 | 1 | 1 | 4 |
| C | 8 | 2 | 5 | 10 | 13 | 16 | 18 | 9 | 9 |
| G | 2 | 4 | 1 | 3 | 1 | 2 | 2 | 1 | 9 |
| T | 19 | 19 | 24 | 13 | 16 | 12 | 10 | 20 | 9 |

The scoring matrix is defined by the following formula [9]:

$s_{i}(a)=\log_{2}\frac{f_{i}(a)+\varepsilon.p(a)}{p(a)(1+\varepsilon)}$

$f_{i}(a)=\frac{f'_{i}(a)}{N_{S}}$

where *p(a)* is the background frequency (take *p(a)=0.25* for the 4 nucleotides), *ε*=0.5 is the Bayesian prior parameter [10] and *N_S_* the number of sequences (in this case 31).

For any *N*-mer we take: $S_{N}=\sum_{i=1}^{N} s_{i}(a)$

Any 9-mer for which S_9_ > 4.5 was considered a putative binding site for the U2AF^65^ subunit.
