# Supplementary material for: Genome-wide identification of functionally distinct subsets of cellular mRNAs associated with two nucleocytoplasmic-shuttling mammalian splicing factors
Source: Genome Biol. 2006 Nov 30;7(11):R113. doi: 10.1186/gb-2006-7-11-r113 (PMC1794580; doi:10.1186/gb-2006-7-11-r113)
Supplement: Additional data file 5 — Primer sequences used. [file gb-2006-7-11-r113-S5.doc]

The sequence of primers used for real time PCR amplification of selected cDNA targets is as follows (forward primer/reverse primer).

**AHSA2**: AATGGAGATGTGGGAACTGG/AAAATGCTGCTTCTGCCAAC;

**APAF1**: GCTCTGCCTTCTCTGTGGACA/GTCAGTCACCCAGCCTCCAT;

**CDKN1B:** GGCCTCAGAAGACGTCAAAC/ACAGGATGTCCATTCCATGA;

**CEP2**: ACGGAGGAAGCTGAAGAGGG/CCTCTTTCTGCAGTTCAGCCA;

**CPSF1**: AAGCCAAGGAGAGTTTCGGG/GGCAAACCACGTGATGTGC;

**CCNL1**: TGCTTGCATCTACCTTGCAG/GGTTGAAAGGGCTGGAGTTC;

**G6PD**: GAACGTGAAGCTCCCTGACG/TCAATCTGGTGCAGCAGTGG;

**GAS2L1**: CCCATGATCAAGGTCTCAGAGG/ TCGTGCTTGTCCAGGTAATGC;

**GLP2R:** AGCCCACAGTGCTTCCTGAG/ TGTTGTCCAGCACCCTGTGT;

**LEPR:** TGCAGGTTTATATGTAATTGTGCCA/CTTCTGAAAATTAAGTCCTTGTGCC;

**MAPK8:** CAGTCAGGCAAGGGATTTGT/TGCTTGTCAGGGATCTTTGG;

**p66alpha**: AGCCCCAGAGATGAACTTCCTG/CATCCTGCCTTGTGTCTCCAGT;

**RAB5C**: CAAGCCTATGCAGACGACAA/AAAGGTGCAGGTGGAATGAC;

**SEC22L3**: CTCAGATGGAGTGCAGCTTGG/TCCATTCGGAAATTAGGAGCAG;

**SMARCA2**: GAAGAGTCAGAGTCCGAGGCA/TCGCTCACTACAGGTTTGGCT;

**SYNGR3**: GGGCTTCTCAGGACTCTGGTC/CCACTGATTGGTGAGGAAGCA;

**U2AF2**: GGGGCTTACCCAACTACCTG/GGTCACAGGCGTCTGATTG;

**ZNF174**:AGAAACCAAAGCAGTGGGTGG/AAGGCTCTGCTGGAGAGCTCT.

Actin intronic and exonic primer pairs were as described in [47].

The following primer pairs were used for amplification of fully spliced mRNAs.

**AHSA2:** TGAGTGGAACATCAAACTGG/TGACACTTTTTCCGCTTACAA

**CDKN1B:** CCTGCAACCGACGATTCTTCT/CCATTCCATGAAGTCAGCGA

**HEXIM:** AAGCGGCATTGGAAACCGTA/AACATCTCGGCTCGGATCCTT

**ZNF174:** AGAAACCAAAGCAGTGGGTGG/CATGGCTTTGCTCCTTTAGC
